# 4343 Utilization of quantitative and qualitative methodology to characterize patient-level factors associated with sustained data transmission and clinical benefit from remote patient monitoring over 12 months

**DOI:** 10.1017/cts.2020.285

**Published:** 2020-07-29

**Authors:** Elizabeth Barnhardt Kirkland, Katie Sterba, Patrick Mauldin, William P. Moran

**Affiliations:** 1 Medical University of South Carolina

## Abstract

OBJECTIVES/GOALS:

1. Identify patient-level factors associated with hemoglobin A1c reduction and sustained device use after 12months of participation in a diabetes and hypertension remote monitoring program
2. Utilize qualitative methodology to characterize key barriers and facilitators to remote monitoring engagement

METHODS/STUDY POPULATION: All participants in statewide quality improvement initiative utilizing a cellular-enabled device with glucose and blood pressure monitoring capability will be included in quantitative analysis (N = 302 at baseline and N = 125 at 6 months at the time of analysis). We developed multilevel regression analyses to model factors associated with clinical outcome (hemoglobin A1c change) and transmission frequency over time. Focus groups and surveys will be conducted to identify barriers and facilitators to continued data transmission and hemoglobin A1c change over 12 months. Semi-structured interview guides are mapped to Wagner’s Chronic Care Model. RESULTS/ANTICIPATED RESULTS: Overall, program participation was associated with 1.8% and 1.3% A1c reduction at 6 (n = 302) and 12 months (n = 125). Regression models showed no association of age, gender, race, income, or insurance with hemoglobin A1c change. Modeling of patient factors associated with sustained transmission frequency or device use is ongoing. Patient focus groups and surveys are currently being scheduled and qualitative data will be analyzed using content analysis. After completing qualitative and quantitative data analyses independently, we will use graphical matrix configurations (“joint displays”) to synthesize findings. DISCUSSION/SIGNIFICANCE OF IMPACT: Our goal is to identify variables associated with the likelihood of patients to engage in and benefit from sustained remote monitoring. Results may inform health policy and guide recruitment approaches, implementation strategies, and methodologic design for future trials. CONFLICT OF INTEREST DESCRIPTION: The authors have no conflicts of interest or disclosures to report

